# Doctors’ perceptions of the impact of upfront point-of-care testing in the emergency department

**DOI:** 10.1371/journal.pone.0208655

**Published:** 2018-12-13

**Authors:** Lara Nicole Goldstein, Mike Wells, Craig Vincent-Lambert

**Affiliations:** 1 Division of Emergency Medicine, Faculty of Health Sciences, University of the Witwatersrand, Johannesburg, South Africa; 2 Department of Emergency Medical Care, Faculty of Health Sciences, University of Johannesburg, Johannesburg, South Africa; University of California at Davis, UNITED STATES

## Abstract

**Objectives:**

Special investigations (e.g. blood tests, electrocardiograms, x-rays) play an integral role in patient management in the emergency department (ED). Having results immediately available prior to assessing a patient may lead to improved efficiency. This could be instituted by utilizing point-of-care (POC) testing with an alternative ED workflow, but the implementation would be dependent on acceptance by the end-users. The aim of this study was to assess doctors’ perceptions of POC testing in the ED when the normal treatment pathway was modified to use upfront POC tests performed prior to doctor evaluation in an effort to decrease treatment times.

**Methods:**

A prospective, randomized, controlled trial was performed in the ED where medical patients received either the normal ED workflow pathway or one of the enhanced workflow pathways with POC tests in various combinations prior to doctor evaluation. At the end of the study period, doctors were invited to participate in an anonymous survey to gauge their opinions on the implementation of the early POC testing.

**Results:**

Overall, the doctors surveyed were very satisfied with use of upfront POC in the ED. One hundred per cent of the 28 doctors surveyed found it helpful to assess patients who already had test results available and would want it to be permanently available. Normalized satisfaction scores were more favorable for combinations of 3 or more tests (0.7–1.0) as opposed to combinations with 2 or less tests (0.3–0.7). There was a preference for combinations that included comprehensive blood results.

**Conclusion:**

The implementation of workflow changes to assist doctors in the ED can potentially make them more productive. End-user buy-in is essential in order for the change to be successful. Upfront, protocolised, POC testing is a low-input, high-yield intervention that decreased treatment time and satisfied doctors.

## Introduction

Time-pressure is ever-present in the emergency department (ED). The need to deliver timeous, quality care and to deal optimally with critically ill and injured patients leads to a hazardous, high density of decision-making [1]. Ways to increase efficiency of ED operations may lead to decreased physician cognitive burden, decreased treatment times and decreased overcrowding, thus having a positive impact on patient care [2].

Laboratory testing and x-rays can be time-consuming, rate-limiting steps for many patients in the ED [3–5]. When doctors have the results of these investigations immediately available when initially assessing a patient, it can decrease treatment times and improve efficiency in the ED [6].

Changes in ED functionality and processes can have an impact on patient flow. However, if an intervention is to be effective, it needs to perform (i.e. deliver efficiency) as well as be supported by the staff who will be using it. Changes will only be effective if they are embraced and adopted by the medical personnel [1]. Successful implementation of a novel system is difficult without acceptance from the end-user.

Until recently, the benefit of Point-of-Care (POC) tests in the ED has only been evaluated *after* the patient has interacted with a doctor or nurse. Protocolised usage of POC tests based on symptom presentation before doctor assessment has just been explored; with promising results [6]. The substantial and significant time-saving produced by this innovation warrants further investigation into identifying barriers to its potential implementation.

The aim of this study was to assess doctors’ perceptions of POC testing when the traditional ED workflow pathway was modified to make use of upfront, POC tests (blood tests, electrocardiograms (ECGs) and/or LODOX (LOw DOse X-ray)) performed prior to doctor evaluation in an effort to decrease treatment times.

## Materials and methods

### Study design and setting

**Fig 1:**
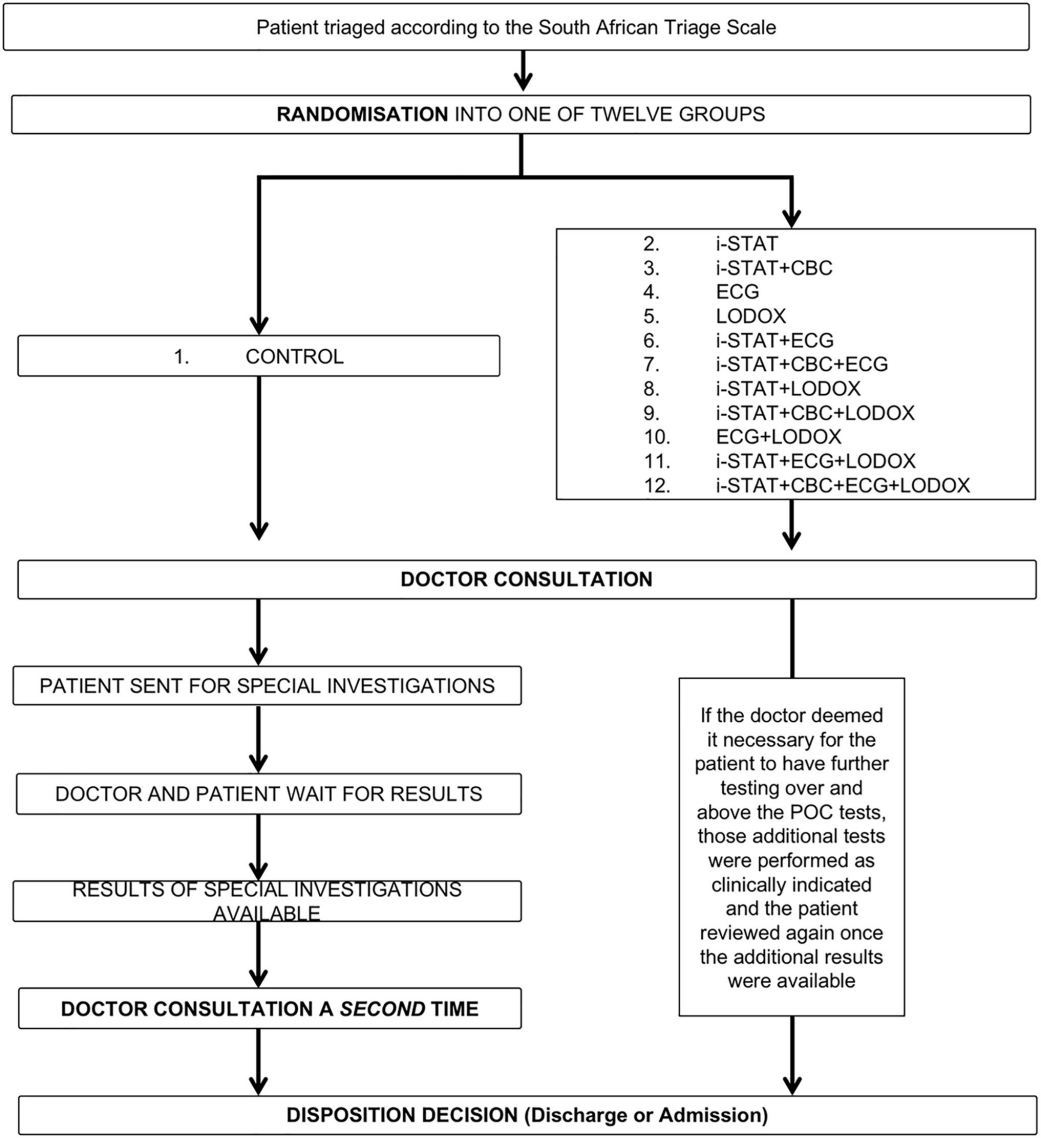
The normal ED patient workflow compared to the POC intervention workflows during the randomized controlled trial period. CBC—Complete Blood Count, ECG—electrocardiogram, i-STAT—i-STAT POC tests, LODOX—LOw-DOse X-ray.

An investigator-initiated, prospective, randomized, controlled trial assessing the utility of upfront, POC tests was conducted in the ED between 13 February and 29 June 2017. Fig 1 demonstrates how the normal ED patient workflow compared to the POC intervention workflows during the randomized, controlled trial period [6]. The annual census for this ED is 65 000 patients. During the final two weeks of patient enrolment (15–29 June 2017), the doctors were invited to participate in a survey to gauge their opinions on the performance and utility of the early POC testing. All doctors working in the ED during this study were eligible for inclusion. There were no exclusion criteria.

Permission to conduct the study was granted by the Research Ethics Committee of the Faculty of Health Sciences of the University of BLINDED (REC-01-185-2016 and REC-01-51-2017); the Human Research Ethics Committee of the University of the BLINDED (M171086) as well as the BLINDED National Health Research Ethics Committee (DOH-BLINDED). Written informed consent was obtained from all doctors who participated in the survey. The randomized controlled trial was registered with clinicaltrials.gov (NCT BLINDED).

### Study questionnaire

An anonymous survey regarding the impact of POC tests on clinical care in the ED was distributed (S1 File). The purpose of the questionnaire was to investigate user perceptions based on their experiences with the POC test results. It was designed to explore their opinions on the operational impact of the tests with regards to time-saving for patients triaged yellow (those to be seen within 1 hour of ED arrival) and orange (those to be seen within 10 minutes of ED arrival) according to the South African Triage Scale as well as for them to rank the individual as well as various combinations of test results that were available during the study.

The POC tests that were evaluated are shown in Table 1.

**Table 1:** POC equipment and tests evaluated in the study.

**Abbott Point-of-Care i-STAT System**
The i-STAT System consists of a handheld POC blood analyzer and single-use i-STAT test cartridges (i-STAT, Abbott Point of Care, Princeton, NJ, USA). The CHEM8+ (sodium, potassium, chloride, total carbon dioxide, ionized calcium, glucose, urea, creatinine, hematocrit, hemoglobin and anion gap), PT/INR (prothrombin time and international normalized ratio), CG4+ (Lactate; pH; partial pressure carbon dioxide (PCO_2_); partial pressure of oxygen (PO_2_); total carbon dioxide; bicarbonate; base excess and oxygen saturation) and Troponin I i-STAT cartridges were utilised on all patients. A venous blood specimen was drawn for this purpose.
**Abbott CEL-DYN Emerald 22 benchtop hematology system**
The CEL-DYN Emerald 22 benchtop hematology system, capable of providing a POC Complete/Full Blood Count (CBC) as well as a white blood cell differential count, was used.
**ECG**
Philips Pagewriter TC30 ECG machines were utilised to obtain the electrocardiograms. A standard 12-lead ECG as well as a right-sided (V1R-V6R) and posterior (V7-V9) ECG were performed on all patients randomized to receive an “ECG”.
**LODOX**
A radiographer performed the LODOX (LOw-DOse X-ray) radiographs (chest and abdomen, antero-posterior and lateral) on a Lodox Xmplar-dr. The radiation exposure was approximately 339uGy per patient versus a standard chest and abdomen radiograph of approximately 5200uGy [7].

### Statistical analysis

The data were analyzed descriptively, with proportions represented by percentages.

To determine the satisfaction score from the respondents’ ranking of the test combinations, seven points were allocated to the most highly-ranked test combination, six points to the next test combination, and so on for each respondent (n = 24). The final satisfaction score was obtained by adding the point allocation from each respondent for each test combination (a maximum score would have been 168). Respondents who did not rank all items (or who did not follow the instructions) were excluded (n = 4).

Due to the small number of doctors, the likert scale was condensed to 3 levels i.e. strongly agree and agree were combined to “(strongly) agree” and strongly disagree and disagree were combined to “(strongly) disagree”.

Data analysis was carried out using SAS (version 9.4 for Windows). The 5% significance level was used.

## Results

### Response rate

The doctors working in the ED ranged in experience from 2 to 5 post-graduate years i.e. medical officer and registrar level. In order to maintain the anonymity offered by the survey, demographic details of the respondents were not sought. There was a 93% response rate– 28/30 eligible doctors completed the survey.

**Fig 2:**
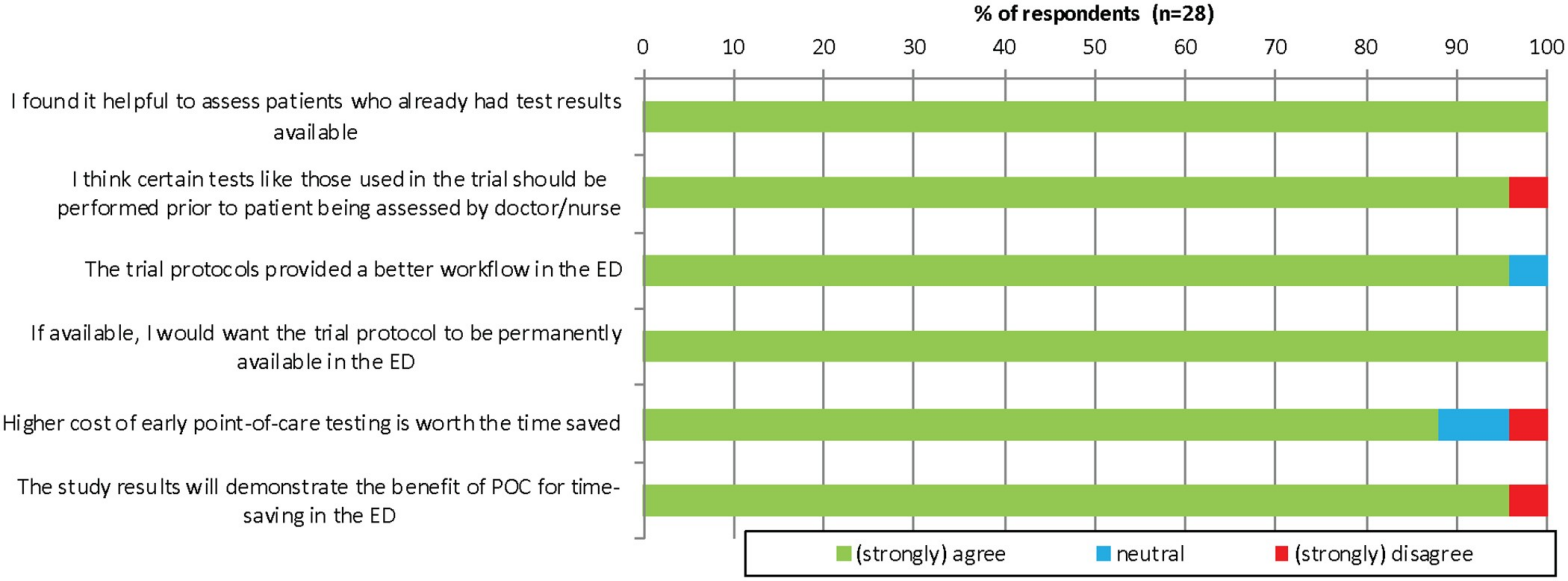
Bar chart of doctors’ likert scale responses to POC testing in the ED.

Overall, the doctors surveyed were very satisfied with use of POC in the ED and would want it to be permanently available (Fig 2).

The normalized satisfaction scores for the individual tests from most to least helpful were i-STAT (1.00); ECG (0.84); CBC (0.48) and LODOX (0.43).

**Fig 3:**
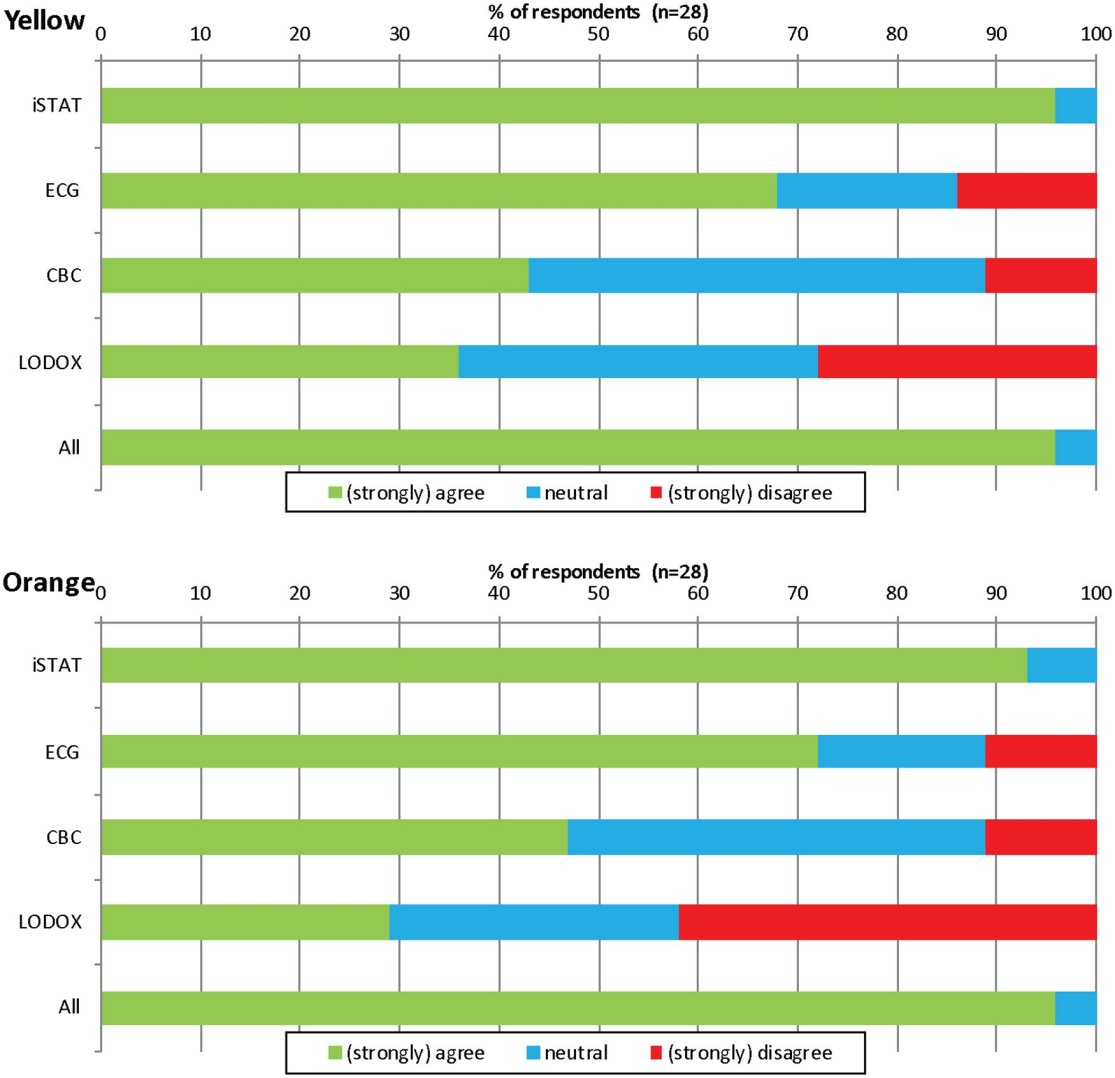
Doctors’ opinion on the time-saving of individual POC tests for patients triaged yellow and orange according to the South African Triage Scale. CBC—Complete Blood Count, ECG—electrocardiogram, i-STAT—i-STAT POC tests, LODOX—Low-dose x-ray.

The doctors’ opinion regarding the perceived time-saving of the separate tests amongst patients triaged yellow (to be seen within 1 hour of ED arrival) and orange (to be seen within 10 minutes of ED arrival) in the ED are shown in Fig 3.

**Fig 4:**
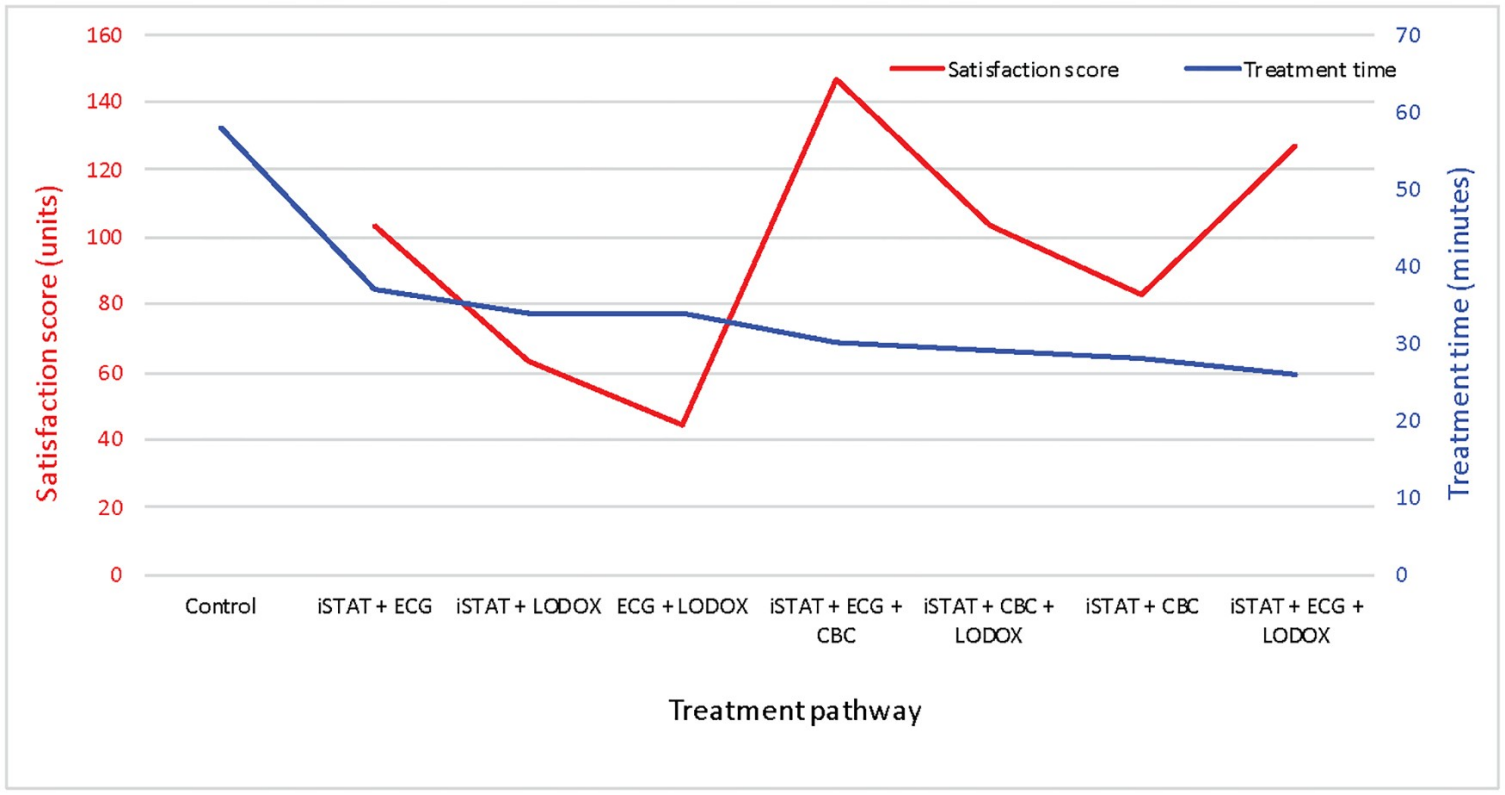
Doctors’ satisfaction with the various POC workflow options compared to treatment time benefits. Actual treatment time for each workflow pathway is indicated in minutes. CBC—Complete Blood Count, ECG—electrocardiogram, i-STAT—i-STAT POC tests, LODOX—Low-dose x-ray.

Fig 4 highlights the doctors perceptions of benefit and satisfaction with the various POC test combinations compared to actual treatment time effects. There was no association between time-saving benefit for the patients and doctors’ satisfaction.

## Discussion

The challenges of working in the ED abound [1]. There is a need to deliver time-critical interventions in an environment where time is elusive and diagnostic uncertainty is pervasive. This makes the exploration of ways to safely expedite patient throughput a priority. However, without the support of these methods by the end-user, implementation is less likely to be successful.

The main outcome measure for the POC study was a decrease in treatment time [6]. The use of upfront POC tests did result in a substantial and significant reduction in treatment times. While decreased treatment times can impact patient satisfaction positively, it can also improve staff satisfaction [4].

### Necessity of special investigations in the ED

As part of the diagnostic workup in the ED, many patients undergo special investigations such as blood tests, ECGs and radiological examinations [8]. These adjunctive tests have a two-fold role—they assist the doctor in confirming or refuting a particular diagnostic hypothesis as well as aiding in the assessment of severity of illness which in the ED is critical to make the disposition decision regarding admission or discharge. Without the delay of waiting for results, the doctors may have preferred having all the information needed to make a diagnosis (history, clinical examination, special investigations) for the patient available at once in order to make the disposition decision quicker and potentially easier. The results could allow rapid identification of patients needing admission thereby avoiding the need for further investigations to take place in the ED.

### Doctors’ attitudes

Similar to the findings in other studies, utilization of POC testing in this study was favorably received by the doctors who felt that its use was associated with expedited care and improved patient flow [2, 9]. The doctors uniformly wanted the POC testing protocol to be implemented in the ED permanently. Their perception of time-saving extended across patients triaged both yellow and orange. They had a preference for test combinations that included comprehensive blood results i.e. i-STAT combined with CBC (Complete Blood Count). When the individual “helpfulness” of the tests was evaluated, the i-STAT alone (which included the 4 tests described in Table 1) was ranked the highest. This may indicate the doctors’ inclination towards more tangible results that require little interpretation compared to the more subjective interpretation associated with either ECGs or x-rays. Blood results may also guide management more definitively. Unless a disorder like an ST-elevation myocardial infarction is diagnosed on ECG or a pneumothorax is diagnosed on x-ray, these modalities are rarely diagnosis clinchers but rather adjuncts.

### Association between attitude and actual outcomes

The lack of association between the satisfaction of the doctors and the actual treatment time benefits of the POC tests was intriguing. Again, having the results immediately available may have fulfilled their preference for irrefutable evidence to confirm a diagnostic hypothesis in an effort to take the uncertainty out of their undifferentiated ED patient. The decrease in treatment time may not have been their primary criterion for liking the POC tests, but rather the POC test ease of availability.

### Barriers to innovation implementation

#### Cost

The costs of POC tests are frequently higher than conventional testing [6, 10]. This was accordingly alluded to in the survey. The possibility of higher costs of the POC tests was a concern and did slightly decrease the doctors’ positivity towards the benefits of the implementation of POC testing. Contrary to this, the test-for-test direct cost comparison discovered in the post-hoc analysis of the costs involved in the POC study surprisingly demonstrated that there was very little cost difference between the POC tests and the traditional options [6]. Similar to risk-benefit ratios, cost considerations do impact decision-making when new system modifications are proposed. Cost of POC may not be the implementation-limiting factor it was originally perceived to be, however.

#### Change management

The survey results, although subjective, are an important part of ensuring the success of a POC protocol in the ED [2]. If the end-user is not happy with the system; its implementation is likely to be difficult. Even the best and most innovative ideas can fail if the participants do not support it. Successful diffusion of quality improvement projects requires leadership and a culture conducive to accepting the change [11]. This change can only work if it takes root in people of the organization [1, 12].

## Limitations

Limitations of this study include that it was performed in a single center making extrapolation of the results to other EDs problematic. There was no assessment of unnecessary testing; although the findings of previous studies have suggested that this was not likely [13, 14]. There was also no assessment of whether upfront POC testing affected patient outcomes.

## Conclusion

According to Richard Branson, “Happy employees equal happy customers” [15]. Likewise, the implementation of systems to assist employees (and ultimately assist our patients) can potentially make them more productive. Upfront, protocolized, POC testing is a low-input, high-yield intervention that not only benefited the patients but also satisfied the doctors.

## Supporting information

S1 File Survey questionnaire on POC testing in the ED. (PDF)

S2 File Excel spreadsheet of raw data. (XLSX)
