# Correction: Doctors' perceptions of the impact of upfront point-of-care testing in the emergency department

**DOI:** 10.1371/journal.pone.0211672

**Published:** 2019-01-29

**Authors:** The PLOS ONE Staff

Several institution names were omitted during peer review and were not replaced before publication. The second paragraph under sub-heading “Study design and setting” in the Materials and methods section should read: Permission to conduct the study was granted by the Research Ethics Committee of the Faculty of Health Sciences of the University of Johannesburg (REC-01-185-2016 and REC-01-51-2017); the Human Research Ethics Committee of the University of the Witwatersrand (M171086) as well as the South African National Health Research Ethics Committee (DOH-27-0117-5628). Written informed consent was obtained from all doctors who participated in the survey. The randomized controlled trial was registered with clinicaltrials.gov (NCT03102216).

Reference 6 was omitted from the article. Reference 6 should read: Goldstein LN, Wells M, Vincent-Lambert C. A randomized controlled trial to assess the impact of upfront point-of-care testing on emergency department treatment time. Am J Clin Path. 2018; 150(3):224–234. doi: 10.1093/ajcp/aqy042

The publisher apologizes for these errors.

## References

[1] Goldstein LN, Wells M, Vincent-Lambert C (2018) Doctors’ perceptions of the impact of upfront point-of-care testing in the emergency department. PLoS ONE 13(12): e0208655 10.1371/journal.pone.0208655 30543668 PMC6292565

