# Supplementary material for: Doctors’ perceptions of the impact of upfront point-of-care testing in the emergency department
Source: PLoS One. 2018 Dec 13;13(12):e0208655. doi: 10.1371/journal.pone.0208655 (PMC6292565; doi:10.1371/journal.pone.0208655)
Supplement: S1 File — (PDF) [file pone.0208655.s001.pdf]

# POC QUESTIONNAIRE

ASSESSMENT OF THE IMPACT OF ENHANCED WORKFLOW PATTERNS ASSOCIATED WITH UPFRONT, EARLY POINT-OF-CARE TESTING ON WAITING AND DISPOSITION TIMES IN AN EMERGENCY DEPARTMENT

***Please circle the option you feel is the most correct***

## 1. Regarding patients seen in *YELLOW MEDICAL AREA*

| | Strongly Disagree | Disagree | Neutral | Agree | Strongly Agree |
|---|---|---|---|---|---|
| It saved time when patients **only** had the **iSTAT (troponin, INR, Chem8, CG4+) results** when seen in *Yellow Medical* | 1 | 2 | 3 | 4 | 5 |
| It saved time when patients **only** had the **CBC (complete blood count) results** when seen in *Yellow Medical* | 1 | 2 | 3 | 4 | 5 |
| It saved time when patients **only** had the **LODOX** when seen in *Yellow Medical* | 1 | 2 | 3 | 4 | 5 |
| It saved time when patients **only** had the **ECG** when seen in *Yellow Medical* | 1 | 2 | 3 | 4 | 5 |
| It saved time when patients had **all EPOC tests** when seen in *Yellow Medical* | 1 | 2 | 3 | 4 | 5 |

## 2. Regarding patients seen in *ORANGE MEDICAL AREA*

| | Strongly Disagree | Disagree | Neutral | Agree | Strongly Agree |
|---|---|---|---|---|---|
| It saved time when patients **only** had the **iSTAT (troponin, INR, Chem8, CG4+) results** when seen in *Orange Medical* | 1 | 2 | 3 | 4 | 5 |
| It saved time when patients **only** had the **CBC (complete blood count) results** when seen in *Orange Medical* | 1 | 2 | 3 | 4 | 5 |
| It saved time when patients **only** had the **LODOX** when seen in *Orange Medical* | 1 | 2 | 3 | 4 | 5 |

| | | | | | |
|---|---|---|---|---|---|
| It saved time when patients **only** had the **<u>ECG</u>** when seen in *Orange Medical* | 1 | 2 | 3 | 4 | 5 |
| It saved time when patients had **<u>all EPOC tests</u>** when seen in *Orange Medical* | 1 | 2 | 3 | 4 | 5 |

## 3. General

| | Strongly Disagree | Disagree | Neutral | Agree | Strongly Agree |
|---|---|---|---|---|---|
| I found it helpful to assess patients who already had test results available | 1 | 2 | 3 | 4 | 5 |
| I think certain tests like those used in EPOC should be performed prior to the patient being assessed by a doctor/nurse | 1 | 2 | 3 | 4 | 5 |
| The trial protocols provided a better workflow in the ED | 1 | 2 | 3 | 4 | 5 |
| If available, I would want the trial protocol to be permanently available in the ED | 1 | 2 | 3 | 4 | 5 |
| Higher cost of early point-of-care testing is worth the time saved | 1 | 2 | 3 | 4 | 5 |

4. Please **RANK** the tests from **MOST helpful (1)** to **LEAST helpful (4)**

| **iSTAT** | **CBC** | **LODOX** | **ECG** |
|---|---|---|---|
| | | | |

5. Please **RANK** the **COMBINATION** of tests from **MOST helpful (1)** to **LEAST helpful (7)**

| **iSTAT + CBC** | **iSTAT + ECG** | **iSTAT + CBC + ECG** | **iSTAT + LODOX** | **iSTAT + CBC + LODOX** | **ECG + LODOX** | **iSTAT + ECG + LODOX** |
|---|---|---|---|---|---|---|
| | | | | | | |

6. **RESULTS PREDICTION**

| | Strongly Disagree | Disagree | Neutral | Agree | Strongly Agree |
|---|---|---|---|---|---|
| The study results will demonstrate the benefit of POC time-saving in the ED | 1 | 2 | 3 | 4 | 5 |

7. **ANY FURTHER COMMENTS OR THOUGHTS?**

______________________________________________

______________________________________________

______________________________________________

______________________________________________

______________________________________________

# THANK YOU FOR YOUR TIME TO COMPLETE THIS SURVEY!
